# Short-term cognitive impacts of electronic gaming machines with and without a skill-based component: A comparative laboratory study

**DOI:** 10.3389/fpsyt.2022.979694

**Published:** 2022-08-26

**Authors:** Sally M. Gainsbury, Kahlil S. Philander

**Affiliations:** ^1^Faculty of Science, School of Psychology, Brain and Mind Centre, University of Sydney, Camperdown, NSW, Australia; ^2^School of Hospitality Business Management, Carson College of Business, Washington State University, Everett, WA, United States

**Keywords:** skill gambling, problem gambling severity, immersion, electronic gaming machines, irrational cognitions

## Abstract

This study aimed to compare how consumers understand the role of skill and chance, experience cognitive distortions, and experience immersion based on use of either electronic gaming machines (EGMs) or skill gambling machines (SGMs; EGMs with a skill-based component). Participants (*N* = 246, *M*_age_ = 34 years, 56.91% female) in a laboratory experiment were randomly assigned to play a real EGM or SGM without funds and self-reported measures including intention to gamble, understanding of the role of skill and chance, erroneous gambling beliefs, previous gambling and gaming, and problem gambling severity. Participants demonstrated different deficits in understanding of the role of skill vs. chance in determining outcomes following play. SGM players were more likely to increase their belief that a skill impacts outcomes and focused more on the game play experience. EGM players focused more on wins and personal performance. Intention to play both machines was predicted in both groups by greater experience of immersion during play and breadth of previous gambling, but breadth of previous gaming experience only predicted intent to play SGMs. The results revealed that both EGM and SGM players fail to understand how outcomes are determined, which is likely more problematic for EGM players as this reflects clear cognitive distortions. Further real-world testing is required to understand the extent to which SGMs harms may be different than EGMs, however, these initial findings suggest that their risks appear comparable to EGMs while attracting individuals with more gaming experience.

## Introduction

The phenomenon of gambling-gaming convergence is increasingly recognized across many channels and products (1). Research has typically approached this convergence by studying the incorporation of gambling elements into gaming products (e.g., loot boxes, skins betting, social casino games). This approach is driven by concerns that gambling-themed games and mechanics may create a pathway to gambling and the development of gambling problems in video game players [e.g., (2–4)].

The gambling industry has contributed to gambling-gaming convergence with the development of skill-based gambling machines (SGMs). SGMs vary in their design elements but are a variant of electronic gaming machines (EGMs; also known as slot/poker machines, video lottery terminals, fixed odds betting terminals) that incorporate skill-based features into chance-based EGMs. SGMs allow skill to influence the house advantage and often incorporate gaming-like themes, but still produce negative expected value outcomes for players.

Despite the popularity of research in gaming products with gambling elements, there is limited study of SGMs. This lab-based study compares generic reel-based EGMs against SGMs, with the aim of investigating the potential market appeal and relevant cognitive and psychological impacts of incorporating skill gaming elements into gambling products. This approach of examining gambling products with gaming components complements the growing literature on gambling-gaming convergence, which has largely focused on gaming products with gambling components. Gambling policy makers and regulators are increasingly focused on ensuring that gambling products do not cause or exacerbate gambling problems (5, 6). Consequentially, it is important to understand whether newly developed gambling products may appeal to vulnerable segments of the population who are at greater risk of experiencing gambling-related harms or exacerbate risk-factors believed to increase risk of harms.

## Background: Electronic gaming machines and skill-based gambling machines

SGMs are designed to appeal to a market segment broader than traditional EGM players, but the extent to which SGMs appeal to individuals in populations vulnerable to gambling problems is an open question. Previous research comparing SGMs and EGMs shows that most participants believe EGMs involve at least some element of skill despite the reality that outcomes are purely chance-based (7, 8) and there are concerns that SGMs emphasis of skill components may exacerbate these effects (9). Cognitive distortions are believed to play a critical role in the development and maintenance of gambling disorders (10–13) and related effects is therefore important to understanding the riskiness of SGMs. Experience with gaming may increase interest in SGMs given familiarity with a gambling modality has been associated with a person feeling overconfident in their ability and likelihood of winning (14, 15).

Some concerns have been raised regarding the extent to which SGMs may contribute to gambling problems through immersive experiences [see (9, 16)]. Video gaming elements of SGMs provide an engaging and immersive experience for players. This state of hyper-focus could persist following cessation of the task, which suggests that immersion decreases one’s ability to re-engage with the “real world” (17). Findings are mixed about whether immersion in gambling is related to escapism from negative emotions or a focus on gambling outcomes (18, 19). Regardless, it can cause individuals to lose track of time and their surrounding environment. This can result in spending more time and money gambling than intended (20). Immersion (sometimes termed “dissociation” in the gambling literature) is argued to be linked to the development and maintenance of gambling problems (21, 22).

Non-randomized studies found that SGMs were more likely to be played by consumers who were younger, had experience playing EGMs and/or mobile games, and had higher problem gambling severity levels (23). Intent to play EGMs and SGMs was predicted by the Theory of Reasoned Action (24), which emphasizes the roles of positive personal attitudes and social norms on future play. We refer to reported intent to play EGMs and SGMs as *intention* to gamble. Intention can be measured by self-report in an experimental setting and has been demonstrated to be closely related to actual future behavior (25, 26).

To expand previously conducted research, we sought to understand three exploratory research questions:

1. Are there differences in beliefs and understanding of how gaming machine outcomes are determined between participants who played an EGM or SGM?
2. Are there differences in the experience of immersion between participants who played an EGM or SGM?
3. What variables predict intention to play EGMs and SGMs following an initial period of play in demonstration mode of each machine?

## Materials and methods

This study took place in a laboratory setting using commercial machines set to “demo” mode. Participants played on the machines without inserting real-world money and there were no real-world monetary outcomes (prizes) involved. Our design provides an opportunity to study the impact of the structural characteristics of each machine type without the confounding factor of random monetary outcomes. We have reported on other aspects of the broader study in separate publications (7).

The study tests outcomes in reported role of skill and chance, cognitive distortions, and immersion as a function of random assignment to an EGM or SGM. We explore intent to play based on those factors, participation breadth and depth variables (9, 23, 27, 28), problem gambling severity (9), and demographic variables including age, gender, and household income.

### Pre-registration

This analysis, including its aims and experimental protocol, were part of a larger pre-registered study on the Open Science Framework (7). The focus of this study was part of the planned exploratory analyses. No changes were made to the pre-registered procedure during or after data collection. All related papers are linked to the pre-registration and do not overlap.

### Sampling strategy and descriptive statistics

As part of the larger study, the sample drew from three target groups within the Australian population: (i) young adults (aged 18–39 years), as this population is a potential target audience for SGMs and is considered vulnerable given relatively high rates of gambling and gaming-related problems; (ii) individuals who play EGMs at least once per month, as such individuals would likely encounter SGMs if they were made available in licensed gambling venues; and (iii) community members with prior EGM gambling experience, given it is important to understand the potential appeal and impact of SGMs on individuals who attend licensed gambling venues and may be interested in SGMs. To participate, respondents had to be at least 18 years of age, Australian residents, and fluent in English.

*A priori* sample size calculations used statistics from prior SGM research (29) as inputs to our power calculation assumptions. We performed a power analysis for an R-squared test in a multiple linear regression with a similar specification to the prior study, estimating a model with one tested covariate and four control variables. Using the study’s pseudo R-squared value of 0.43, assuming that the variable will add 0.02, and specifying an α of 0.05 and 80% power, a sample of 218 individuals was required. Further details are provided in the pre-registration documents.

A total of 246 participants completed the study. The study sample self-reported as 56.91% female, 42.68% male, and 0.41% other gender. On average, the sample was 34 years old (*SD* = 17.32). Most of the sample were either students or in the workforce: employed full-time (24.39%) or part-time (22.36%); students (39.43%); retired or voluntarily inactive (7.32%); unemployed (3.25%); and other (3.25%). The median reported household income band was AU $78,000–$90,999 per year. Using the problem gambling severity index (PSGI) (30), participants were categorized as mostly experiencing no or low-risk gambling problems (41.87 and 34.15%, respectively) with smaller but notable proportions reporting moderate-risk (12.60%) or severe gambling problems (11.38%). Table 1 provides summary statistics by assignment condition.

**TABLE 1:** Summary statistics by assignment condition.

	Played EGM				Played SGM			
	Mean	SD	Min	Max	Mean	SD	Min	Max
Age	33.72	16.64	18.00	73.00	35.01	18.28	18.00	75.00
Gender*	0.43	0.51	0.00	2.00	0.44	0.50	0.00	1.00
Household income category	8.56	5.42	1.00	16.00	8.23	5.32	1.00	16.00
PGSI score	2.53	3.30	0.00	18.00	2.37	3.45	0.00	18.00
Observations	122				117			

*Female, 0; Male, 1; Other, 2. One respondent in the EGM assignment condition identified as “Other.”

### Recruitment

Young adults were recruited *via* an online research participant recruitment platform hosted by the School of Psychology at the lead author’s institution. This platform allows students to sign up to participate in research studies as part of a voluntary research participation assessment component in exchange for course credit. Students outside of the research participation assessment scheme can also sign up to participate in studies and are offered a monetary reimbursement for their time. Regular EGM players were recruited by distributing leaflets in a local licensed gambling venue and by posting a recruitment notice in an e-newsletter distributed to club members. Additional participants who reported playing EGMs at least monthly were recruited through a recruitment agency. Community members were recruited through word-of-mouth and social media posts, and *via* a recruitment agency.

### Gambling stimuli

The gaming machines used for this study were provided by an Australian gaming device manufacturer. In accordance with Section 8(2) of the *Gaming Machines Act 2001*, approval to use the gaming machines for research purposes was gained from the state gambling regulatory authority prior to commencing the study. Two types of machines were used: (i) a standard reel-based EGM, which was legally available in licensed gambling venues at the time of conducting the present study; and (ii) an EGM with reel spins and a skill-based video-gaming style feature and video-game controller (SGM), which was not available to local consumers at the time of the study. In contrast to the EGM (on which outcomes are determined completely by chance), outcomes on the SGM incorporate both chance and skill elements: the reel-based component is entirely chance-based, but outcomes in the gaming component are fully based on a player’s skill. The bonus round was a third-person-style fantasy game that used separate controls that were similar to a video game controller. In-game currency is awarded to enable the player to improve their account’s in-game content. Nonetheless, all SGM players, regardless of skill, have the same rate of return to player (RTP). More skilled players will yield more in-game assets, but cash win mechanics are unrelated to skill. This means that over a long period of time, all SGM players have the same chances of winning small and large prizes. Both machines were set to return to players a minimum percentage of 90.5%, which is consistent with machine configurations found in real-world gambling venues. Gaming credits were pre-loaded so money was not required to initiate play and participants were not awarded any monetary wins acquired during play.

### Laboratory testing procedure

Ethical aspects of this study were approved by the lead author’s institutional Human Research Ethics Committee (project number 2019/738) prior to commencing data collection. Participants provided informed consent by reading the Participant Information Statement and submitting a signed consent form prior to commencing the pre-test questionnaire.

Up to six participants were included in each session. Upon arrival, consenting participants completed pre-test questionnaires using tablet devices. Once all participants had completed the pre-test questionnaire, they were taken by a researcher to a room housing three EGMs and three SGMs. The researcher instructed each participant to sit at the specific gaming machine corresponding to the experimental condition to which they had been randomly assigned. Participants were instructed to play the machines for 20 min. If after 10 min the game feature had not naturally occurred, the researcher manually triggered the feature (*via* back-end activation) to ensure it was experienced by all participants. The EGM had a traditional free game feature in which participants can choose “red” or “black” on a card flip. The SGM’s third-person-style game was a longer bonus round where the player’s character would fight enemy characters using weapons and spells. Participants were then asked to complete the post-test questionnaire using tablet devices. Once all participants had completed the post-test questionnaire, the researcher provided a verbal debrief to ensure that participants understood the experimental protocol, and the true role of skill and chance in determining outcomes in each machine type.

### Measures

#### Demographic information (pre-test)

Participants were asked to report their gender, age, highest level of education completed, current employment status, country of birth, primary language spoken at home, and total annual household income before tax. Items and response options were adapted from the Australian Bureau of Statistics (31) census survey.

#### Gambling involvement (pre-test)

Participants reported how often they spent money on eight different types of gambling activities in a typical month: instant scratch tickets or lottery games; bingo or keno; private betting (e.g., playing cards or mah-jong with friends and family); poker; casino table games (e.g., blackjack, roulette); poker/slot machines (EGMs); betting on horse/dog races; and betting on sports (32). Responses were measured on a 5-point scale (*not at all* = 0; *1–3 times per month* = 2; *once a week* = 4; *several times a week* = 12; *daily* = 30). Two derivative variables were developed: (1) gambling breadth (a scale ranging from 0 to 8), which is the count of the number of gambling activities on which an individual reported spending money (*M* = 2.52, *SD* = 2.29); and (2) gambling depth (a scale ranging from 0 to 30), which is the highest level of involvement in any single form of gambling (*M* = 4.96, *SD* = 7.00).

#### Gaming involvement (pre-test)

Four questions assessed gaming involvement in relation to frequency across different gaming platforms. Monthly frequency of video game play across four platform types (console games, such as Xbox, Nintendo, PlayStation; mobile phone games; PC/computer games; arcade games) was assessed using a 4-item scale adapted from Delfabbro et al. (33). Responses were measured on a 5-point scale (*not at all* = 0; *1–3 times per month* = 2; *once a week* = 4; *several times a week* = 12; *daily* = 30). Two derivative variables were developed: (1) gaming breadth (a scale ranging from 0 to 4), which is the count of the number of gaming activities on which an individual reported non-zero participation (*M* = 1.88, *SD* = 1.34); and (2) gaming depth (a scale ranging from 0 to 30), which is the highest level of involvement in any single form of gaming (*M* = 11.07, *SD* = 11.78).

#### Gambler’s beliefs questionnaire (pre-test/post-test)

The 21-item Gamblers’ Beliefs Questionnaire [GBQ; (34)] is a widely used psychometric measure of gambling-related cognitive distortions consisting of two subscales: luck/perseverance (13 items) and illusions of control (8 items). Responses are assessed on a 7-point Likert-type scale (*strongly agree* = 1; *strongly disagree* = 7), reverse-coded, and summed to yield an overall score. Higher scores indicate higher levels of gambling-related cognitive distortions. The GBQ demonstrated excellent internal consistency in this sample (Cronbach’s α = 0.91). Respondents were administered the GBQ both before (pre-GBQ) and after (post-GBQ) the experiment.

#### Erroneous estimates of winning (pre-test/post-test)

Participants completed the Erroneous Estimates and Irrational Beliefs Questionnaire (35) both before and after machine play. The four items assess participants’ estimates about the chances of different outcomes when playing EGMs: winning; losing; breaking even; and winning the maximum prize. Responses are measured by participants indicating their responses on a rating scale from 0 to 100%.

#### Intention to gamble on gaming machines (post-test)

Participants indicated their agreement with two items regarding their intentions to gamble on the machine they played on in the next year, assuming such machines were accessible (29), “I want to gamble on the machine I just played on in the next year,” and “There is a chance I could gamble on the machine I just played on in the next year” (*strongly disagree* = 1, *strongly agree* = 5). Participants also responded to one question adapted from experiment 3 of Jennett et al. (17), “Would you like to play the game again?” (*definitely not* = 1; *definitely yes* = 5). The items demonstrated good internal consistency (Cronbach’s α = 0.86).

#### Problem gambling severity (post-test)

The 9-item PSGI (30) is a reliable and accurate screen for presence and severity of gambling problems. Respondents indicate frequency of experiencing symptoms of problem gambling during the past 12 months on a 4-point scale (*never* = 0; *almost always* = 3). Items are summed, and overall scores are classified as follows: non-problem gambling (score of 0); low-risk gambling (score of 1 or 2); moderate risk gambling (score between 3 and 7); and problem gambling (score of 8 or more).

#### Immersion (post-test)

The 31-item immersion questionnaire from experiment 3 of Jennett et al. (17) was used to measure participants’ experience of immersion in the machine play session. Immersion is examined across five factors: cognitive involvement, real world dissociation, challenge, emotional involvement, and control. Participants were instructed to respond to the questions on a 5-point scale (*not at all* = 1; *a lot* = 5) based on how they felt at the end of the game. After reverse-coding six items, item scores are summed to yield a total immersion score. Internal consistency was excellent (Cronbach’s α = 0.94).

#### Understanding of gaming machine outcomes (post-test)

Participants indicated their agreement with ten statements about the way outcomes are determined on the type of machine they played. Eight items assessed understanding regarding the short- and long-term outcomes and profitability of machine play on a 5-point Likert scale (*strongly disagree* = 1; *strongly agree* = 5). The remaining two items assessed understanding regarding the influence of skill vs. chance on outcomes, and the extent of a player’s control over game outcomes. We replicate the summative score variables for SGM and EGM game understanding from Philander and Gainsbury (8).

### Statistical analysis

First, we tested for differences in individual items from the understanding of gaming machine outcome variables (assessed after using the machines). We estimated two-sample Welch’s *t*-tests for each variable based on the SGM/EGM assignment condition. Since this analysis is exploratory and not confirmatory, we did not adjust critical values for multiple pairwise tests.

Second, we tested measures related to beliefs about the role of skill and control on EGMs and SGMs. We focused on those beliefs because skill and control characterize the primary design difference between EGMs and SGMs. Both the Erroneous Estimates and Irrational Beliefs Questionnaire and the Gamblers’ Beliefs Questionnaire assess multiple dimensions of subjective beliefs and cognitive distortions that extend beyond skill and control. We reviewed each of the items in both scales for mention of skill or control. The two items from the Erroneous Estimates and Irrational Beliefs Questionnaire (35) that relate to skill and control:

1. *How much control do you think that you have over the outcome of a game on an EGM?* (IBQ1; scale ranging from 0 to 100)
2. *How much do you think a player’s skill impacts on the outcome of a game on an EGM?* (IBQ2; scale ranging from 0 to 100)

The four items from the Gamblers’ Beliefs Questionnaire (34) that relate to skill and control:

1. *My knowledge and skill in gambling contribute to the likelihood that I will make money.* (GBQ1; *strongly disagree* = 1; *strongly agree* = 7)
2. *My choices or actions affect the game on which I am betting.* (GBQ2; *strongly disagree* = 1; *strongly agree* = 7)
3. *My gambling wins are evidence that I have skill and knowledge related to gambling*. (GBQ3; *strongly disagree* = 1; *strongly agree* = 7)
4. *I have more skills and knowledge related to gambling than most people who gamble*. (GBQ4; *strongly disagree* = 1; *strongly agree* = 7)

Repeated measures were taken before and after the simulated gambling session. Since GBQ3 asks respondents about wins and the respondent may have randomly won or loss based on the short trial period, it was excluded from the analysis. We estimated a principal factor analysis with the remaining variables to assess whether they loaded onto the same factor. As shown in Table 2, all the items except for GBQ4 had acceptably large factor loadings onto Factor 1 (36). We therefore dropped GBQ4 from the analysis. Removing GBQ4 increased Cronbach α of the included items from 0.71 to 0.75.

**TABLE 2:** Factor loadings (pattern matrix) and unique variances.

Variable	Factor 1	Factor 2	Uniqueness
GBQ1	0.64	0.22	0.54
GBQ2	0.53	0.22	0.66
GBQ4	0.24	0.25	0.88
IBQ1	0.67	−0.26	0.48
IBQ2	0.75	−0.20	0.40

We used Welch’s *t*-tests to measure differences in responses before the simulated gambling session and after the simulated gambling session, with the two-samples grouped by the SGM/EGM assignment condition.

Third, we tested for differences in game immersion after play using immersion scale items. We estimated two-sample Welch’s *t*-tests for each variable, based on the SGM/EGM assignment condition. Since this analysis is exploratory and not confirmatory, we did not adjust critical values for multiple pairwise tests.

Fourth, we explored differences in intention to play the gambling devices using a stepwise modeling approach that enables us to consider differences in correlates across a wide range of plausible behavioral and psychometric correlate measures. Since the intention factor variable is non-categorical, we estimated a forward selection stepwise OLS model, with an α = 0.05 significance level for addition to the model. Included as potential variables were gambling depth, gambling breadth, gaming depth, gaming breadth, PGSI score, immersion, pre-GBQ, understanding of gaming machine outcomes, age, gender, and household income. Since one of the items from the immersion scale is used as an item in the intention factor, we removed the item from the immersion scale to avoid a tautological result.

All analyses were estimated using Stata 15.

## Results

### Understanding of skill and chance

**FIGURE 1:**
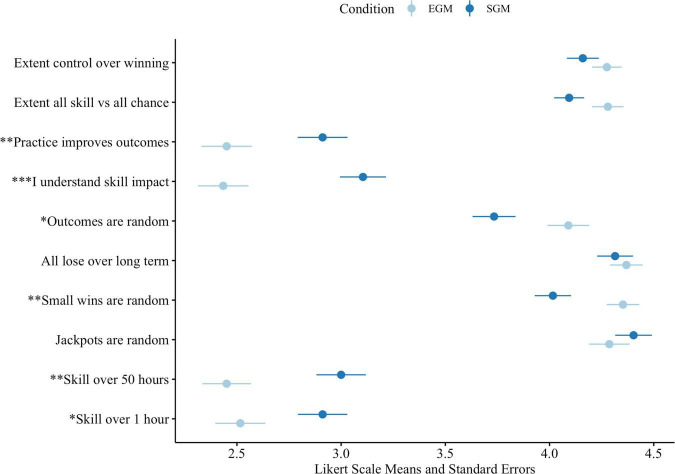
Game understanding item mean responses based on EGM/SGM assignment condition. *p < 0.05, **p < 0.01, ***p < 0.001.

In Figure 1, we illustrate the results of our *t*-tests of the post-session game understanding items across the EGM and SGM assignment conditions. Full results are provided in Supplementary Appendix 1. We find that individuals assigned to the EGM are more likely to somewhat disagree that they understand how a player’s skill impacts the outcomes, that players can improve their outcomes with practice, and that a player of greater skill is more likely to win money over the short and longer term, compared to players assigned to SGMs. SGM-assigned players were more likely to provide neutral responses, indicating a lack of agreement or disagreement with these statements. EGM players had stronger agreement that small in-game wins and the game outcomes are randomly determined than SGM players. Both groups similarly tended to agree that jackpot wins are randomly determined and that over the long term all players will lose money. Further, there was no significant difference in responses based on EGM vs. SGM players that winning money over 50 h of play is based more on chance, and that players have a small amount of control over the outcome of winning money.

### Change in beliefs about the role of skill and control on electronic gaming machines

Some changes in beliefs about the role of skill and control were observed after the experiment and these changes differed based on the assignment condition. Individuals assigned to EGMs had no change in their reported control over the machine outcome (IBQ1: *M* = −0.25, *SD* = 1.90). Individuals assigned to SGMs had an increase in their reported control over the machine outcome (IBQ1: *M* = 0.40, *SD* = 2.07). The groups showed a statistically significant difference in their mean responses, *t*(235) = −2.55, *p* = 0.01.

Individuals assigned to EGMs had a decrease in their reported belief that a player’s skill impacts on the outcome of a game (IBQ2: *M* = −0.51, *SD* = 2.46), whereas individuals assigned to SGMs showed an increase (IBQ2: *M* = 0.36, *SD* = 1.89). The groups showed a statistically significant difference in their mean responses, *t*(228) = −3.07, *p* < 0.01.

Individuals assigned to EGMs had a decrease in their reported agreement that their knowledge and skill in gambling contribute to their likelihood of making money (GBQ1: *M* = −0.75, *SD* = 1.73). Individuals assigned to SGMs had no change in their reported values (GBQ1: *M* = −0.21, *SD* = 1.41). The groups showed a statistically significant difference in their mean responses, *t*(233) = −2.66, *p* < 0.01.

Individuals assigned to EGMs had no change in their reported agreement that their choices or actions affect the game on which they are betting (GBQ2: *M* = −0.22, *SD* = 2.11). Individuals assigned to SGMs also had no change (GBQ2: *M* = −0.28, *SD* = 2.03). The groups showed no difference in their mean responses, *t*(239) = 0.23, *p* < 0.82.

**FIGURE 2:**
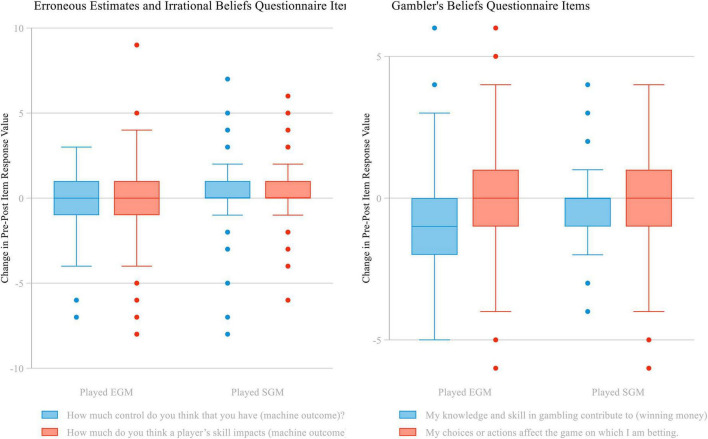
Boxplot of *t*-test data regarding change in beliefs about the role of skill and control on electronic gaming machines.

We observe statistically significant differences in both items from the Erroneous Estimates and Irrational Beliefs Questionnaire (see Figure 2). Individuals assigned to SGMs have greater beliefs that they have more control and more skill-related impact on outcomes compared to individuals assigned to EGMs. We also observe a statistically significant difference in one of the GBQ items. Individuals assigned to SGMs have greater beliefs that their skill contributes to winning.

### Immersion

**FIGURE 3:**
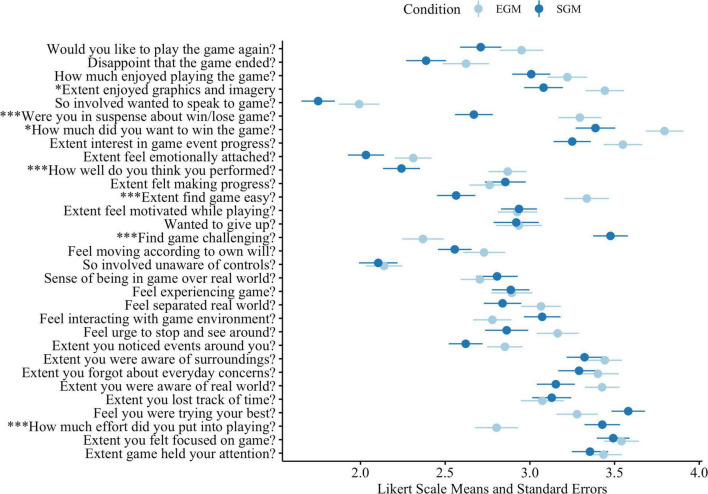
Immersion questionnaire item mean responses based on EGM/SGM assignment condition. *p < 0.05, **p < 0.01, ***p < 0.001.

As shown in Figure 3, our tests of immersion items showed statistically significant differences between EGM and SGM assignment conditions. Compared to individuals assigned to EGMs, SGM players found the game less easy, more challenging, and felt that they put more effort into the game. Compared to individuals assigned to SGMs, EGM players enjoyed the graphics and imagery more, were more in suspense, wanted to win more, and thought they performed better. There were no differences between the groups at the factor level for the majority of immersion items and no clear differences based on subscales with challenge and cognitive dissociation items reported by both groups, and no differences in items for the real world dissociation or emotional involvement items. Full results of the tests are provided in Supplementary Appendix 2.

### Intention to play

Our stepwise model produced a small number of variables that predicted intention to play the game in the future. Results are displayed in Table 3. Both models reported immersion as a predictive variable, which may be a result of the importance of immersion to attitudes in a machine play task that did not involve any value at risk. Interestingly, both models produced gambling breadth as a significant variable but only the SGM model produced gaming breadth as a significant variable. This suggests that individuals who play more gambling types have higher intention to play both games, but individuals who play more gaming types only have higher intention to play SGMs but not EGMs in our study.

**TABLE 3:** Results of stepwise OLS regression predicting intention to play.

Variable	Played EGM	Played SGM
	
	DV: Intention factor	DV: Intention factor
Immersion scale	0.0249***	0.022***
Gambling breadth	0.116**	0.086**
Gaming breadth		0.136**
Constant	−2.293***	−2.595***
*N*	122	117
Adj. *R*^2^	0.538	0.466

Included as potential variables were gambling depth, gambling breadth, gaming depth, gaming breadth, PGSI score, immersion, GBQ (prior to play), understanding of gaming machine outcomes, age, gender, and household income.

*p < 0.05, **p < 0.01, ***p < 0.001.

## Discussion

This study investigated factors predicting intention to gamble on traditional reel-based EGMs and SGMs with a reel and separate skill feature. In addition, we investigated immediate post-play effects on self-reported immersion, cognitive distortions, and understanding of the relative roles of skill vs. chance in determining game outcomes.

Perceptions of the role of chance and skill were influenced by the machine type individuals played. This finding suggests that SGMs and EGMs have differing structural characteristics that may impact understanding of machines and cognitive distortions. Overall, SGM players appeared to be uncertain about the role of skill in determining outcomes, including whether practice can improve outcomes. Although EGM players indicated outcomes are somewhat determined by chance, the responses of participants after playing these machines did not demonstrate a definitive understanding that skill has no role. We observed a similar perceived lack of understanding of skill vs. chance. This is a concern given that skill plays no role at all in EGMs. The results revealed that both EGM and SGM players fail to understand how outcomes are determined, which is likely more problematic for EGM players as this reflects clear cognitive distortions.

Our research provides novel findings about structural characteristics to which players have not been previously exposed. We observed that EGM player perception about the role of skill and chance stayed relatively stable before and after a session of play: views on the impact of player actions and control over machine outcomes were stable, although there was a decrease in perception of skill on outcomes. In contrast, SGM play resulted in increases in perception of reported control and impact of skill on the outcomes, but not in impact of skill or choices on monetary outcomes. These results demonstrate that the structural design and experience of games can immediately impact cognition. Future research is needed to examine the extent to which changes remain after sessions, are strengthened across sessions, and may be specific to experiences with individual products or brands.

We did not find any indication of significant differences in the experience of immersion immediately following EGM or SGM play, particularly in relation to real world or emotional dissociation (e.g., losing track of time, awareness of everyday concerns and surroundings, or engagement in the game). This is somewhat inconsistent with focus groups which indicated that the skill feature of SGMs was more engaging and immersive than EGM reel-spins (37). However, our results did show that SGM players reported putting more effort into the game and that it was challenging (and less easy). EGM players were more focused on outcomes and wins. Although these findings are limited due to the laboratory setting and lack of real money wagered, they provide an initial indication that engagement in the skill aspect of SGMs does not result in any significant changes beyond what may be observed in standard EGM play that would contribute to ongoing or persistent gambling and potential harmful consequences.

Higher immersion scores did predict whether individuals would be likely to play EGMs and SGMs in the future, indicating that this may be a desirable state or outcome following a session of play. Breadth of gambling experience predicted intent to play both machines. This may indicate that if introduced, SGMs would be popular with existing gambling customers rather than relying on novel customers who do not play EGMs. However, as mentioned, experience with gaming appears to impact the likelihood of interest in SGMs, supporting the potential consumer market for these games which includes overlap as well as distinction from existing gambling consumers. There was no indication that individuals potentially vulnerable to experiencing gambling problems, including problem gambling severity score, existing high gambling-related cognitive distortions, or understanding of how outcomes are determined impacted the likelihood that participants would play either machine in the future.

### Limitations and future research

The composition of our sample affects how the study results should be interpreted. The large student cohort conflates young age with other characteristics that might be relevant to findings. For example, young students from a nationally top-performing university are more likely to have above average intelligence and be more career-focused. The community sample of non-students were recruited because of their past involvement with gambling machines. That is, the community sample had an additional screening criterion (prior machine gambling involvement) so at the outset, we may find that older gamblers are more involved in machine gambling, which relates to recruitment methods rather than population-wide behavior. As we randomly assigned individuals in our experiment, this consideration is less relevant to the experimental findings than to the correlational analyses. A strength of this study is that multiple recruitment methods were used to ensure a diverse sample in terms of age and previous engagement with gambling and gaming. Statistically, we treated regression variables as continuous in a linear model, which may not reflect their underlying relationship. All participants were exposed to SGMs for the first time as they were not available in Australia at the time of the study. This provides some control for past SGM experience.

Other limitations include the use of machines without the risk of money or possibility of monetary outcomes, which likely limit engagement with the game through low ecological validity. Only one type of EGM and SGM were used, limiting the ability to extrapolate to other machine designs, which are highly varied for SGMs. We relied on self-report measures, so future studies should use implicit measures of cognition and relevant behavioral measures. Where possible, more realistic testing scenarios, including controlled trials in gambling venues, will provide further important findings related to SGM appeal and impact. Trials are needed to examine medium and long-term impacts after the initial novelty of a newly introduced gambling product reduces.

Individuals assigned to SGMs had greater beliefs that their skill contributes to winning, which is true regarding in-game items but over the long-run all players have a negative expected cash value with return to players set to 90.5%. Accordingly, it may be appropriate for future studies to validate the extent to which common measures of gambling distortions are effective scales for SGM users.

## Conclusion

Consideration of structural characteristics is important when assessing the potential of a gambling activity or product to contribute to harms, including the mechanisms and nature of impacts for individuals currently experiencing or at risk of developing gambling problems. Laboratory studies are an important test of a new gambling product to identify any immediate potential for gambling harms before a product is licensed, even in a limited capacity for real gambling. Our lab-based study found that the experience of in-session immersion and higher levels of previous gambling predict intention to play EGMs and SGMs. Previous gaming experience additionally predicts likelihood to play SGMs. There was no evidence that EGM or SGM play resulted in differential levels of immersion immediately following the session of play. However, the session did impact perceptions of skill and chance, showing that SGM players can perceive a role of skill in the new machines, although they are relatively uncertain about the extent to which this influences outcomes as responses to the item, “I understand how a player’s skill impacts outcomes,” fell near the mid-point of the agreement/disagreement Likert scale at a mean of 3.105.

This paper represents an important contribution to the literature as it reports on the immediate impact of novel structural features of an EGM with an incorporated skill component. As the gambling industry increasingly incorporates gaming aspects into gambling products, ongoing research is needed to provide a strong evidence base to inform responses by regulators and policy-makers. *In situ* testing is needed to understand which players are likely to engage with SGMs in their various configurations immediately and over time. Greater understanding is also needed about the impact of these machines on cognitive and behavioral factors that may impact the experience of gambling problems. In line with a proposed framework for addressing emerging technology with potential harmful consequences (38), we recommend that researchers, industry, government, and community stakeholders collaborate to investigate further, and make proactive efforts to prevent and minimize any potential problems.

## Data availability statement

The original contributions presented in this study are included in the article/Supplementary material, further inquiries can be directed to the corresponding author/s.

## Ethics statement

The studies involving human participants were reviewed and approved by the University of Sydney Human Research Ethics Committee (project number: 2019/738). The patients/participants provided their written informed consent to participate in this study.

## Author contributions

SG led the recruitment and data collection. KP led the data analysis. Both authors contributed to the design and writing of this study.
