# Supplementary material for: Short-term cognitive impacts of electronic gaming machines with and without a skill-based component: A comparative laboratory study
Source: Front Psychiatry. 2022 Aug 26;13:979694. doi: 10.3389/fpsyt.2022.979694 (PMC9462663; doi:10.3389/fpsyt.2022.979694)
Supplement: Supplementary file 2 [file Data_Sheet_2.docx]

# Appendix A: T-tests using Welch’s approximation

Game understanding item differences in EGM and SGM assigned players

|  | Played EGM | Played SGM | Difference | t | p |
| --- | --- | --- | --- | --- | --- |
| ‘A player of greater skill is more likely to win money over 1 hour of play, compared to a player of lesser skill.’ | 2.516 | 2.911 | -0.395 | -2.342 | 0.020 |
| ‘A player of greater skill is more likely to win money over 50 hours of play, compared to a player of lesser skill.’ | 2.451 | 3.000 | -0.549 | -3.306 | 0.001 |
| ‘Jackpot wins (i.e., large sums of money) are randomly determined regardless of skill.’ | 4.287 | 4.403 | -0.116 | -0.889 | 0.375 |
| ‘Small in game wins (i.e., winning a small amount of money in a single game) are randomly determined regardless of skill.’ | 4.352 | 4.016 | 0.336 | 2.875 | 0.004 |
| ‘Over the long term, all players will lose money.’ | 4.369 | 4.315 | 0.054 | 0.470 | 0.639 |
| ‘The outcomes are random no matter what a player does.’ | 4.090 | 3.734 | 0.356 | 2.479 | 0.014 |
| ‘I understand how a player’s skill impacts outcomes.’ | 2.434 | 3.105 | -0.670 | -4.095 | 0.000 |
| With practice a player can improve their outcomes over time.’ | 2.451 | 2.911 | -0.460 | -2.707 | 0.007 |
| ‘To what extent do you think that winning money over 50 hours of play is based on skill or chance?’ | 4.280 | 4.094 | 0.186 | 1.787 | 0.075 |
| ‘To what extent does a player have control over the outcome of winning money?’ | 4.275 | 4.160 | 0.115 | 1.106 | 0.270 |

^*^ *p* < 0.05, ^**^ *p* < 0.01, ^***^ *p* < 0.001

# Appendix B: T-tests using Welch’s approximation

Immersion questionnaire item differences in EGM and SGM assigned players

|  | Played EGM | Played SGM | Difference | t | p |
| --- | --- | --- | --- | --- | --- |
| IMQ1: To what extent did the game hold your attention? | 3.434 | 3.355 | 0.080 | 0.525 | 0.600 |
| IMQ2: To what extent did you feel you were focused on the game? | 3.541 | 3.492 | 0.049 | 0.350 | 0.727 |
| IMQ3: How much effort did you put into playing the game? | 2.803 | 3.427 | -0.624 | -3.800 | 0.000 |
| IMQ4: Did you feel that you were trying you best? | 3.279 | 3.581 | -0.302 | -1.918 | 0.056 |
| IMQ5: To what extent did you lose track of time? | 3.074 | 3.129 | -0.055 | -0.322 | 0.748 |
| IMQ6: To what extent did you feel consciously aware of being in the real world whilst playing? | 3.426 | 3.153 | 0.273 | 1.800 | 0.073 |
| IMQ7: To what extent did you forget about your everyday concerns? | 3.402 | 3.290 | 0.111 | 0.638 | 0.524 |
| IMQ8: To what extent were you aware of yourself in your surroundings? | 3.443 | 3.323 | 0.120 | 0.821 | 0.413 |
| IMQ9: To what extent did you notice events taking place around you? | 2.852 | 2.621 | 0.231 | 1.603 | 0.110 |
| IMQ10: Did you feel the urge at any point to stop playing and see what was happening around you? | 3.164 | 2.863 | 0.301 | 1.695 | 0.091 |
| IMQ11: To what extent did you feel that you were interacting with the game environment? | 2.779 | 3.073 | -0.294 | -1.888 | 0.060 |
| IMQ12: To what extent did you feel as though you were separated from your real-world environment? | 3.066 | 2.839 | 0.227 | 1.410 | 0.160 |
| IMQ13: To what extent did you feel that the game was something you were experiencing, rather than something you were just doing? | 2.893 | 2.887 | 0.006 | 0.039 | 0.969 |
| IMQ14: To what extent was your sense of being in the game environment stronger than your sense of being in the real world? | 2.705 | 2.806 | -0.102 | -0.610 | 0.543 |
| IMQ15: At any point did you find yourself become so involved that you were unaware you were even using controls? | 2.139 | 2.105 | 0.035 | 0.222 | 0.825 |
| IMQ16: To what extent did you feel as though you were moving through the game according to you own will? | 2.730 | 2.556 | 0.173 | 1.087 | 0.278 |
| IMQ17: To what extent did you find the game challenging? | 2.369 | 3.476 | -1.107 | -6.935 | 0.000 |
| IMQ18: Were there any times during the game in which you just wanted to give up? | 2.934 | 2.919 | 0.015 | 0.079 | 0.937 |
| IMQ19: To what extent did you feel motivated while playing? | 2.926 | 2.935 | -0.009 | -0.059 | 0.953 |
| IMQ20: To what extent did you find the game easy? | 3.336 | 2.565 | 0.772 | 4.462 | 0.000 |
| IMQ21: To what extent did you feel like you were making progress towards the end of the game? | 2.762 | 2.855 | -0.093 | -0.547 | 0.585 |
| IMQ22: How well do you think you performed in the game? | 2.869 | 2.242 | 0.627 | 3.999 | 0.000 |
| IMQ23: To what extent did you feel emotionally attached to the game? | 2.311 | 2.032 | 0.279 | 1.839 | 0.067 |
| IMQ24: To what extent were you interested in seeing how the game’s events would progress? | 3.549 | 3.250 | 0.299 | 1.881 | 0.061 |
| IMQ25: How much did you want to ‘‘win’’ the game? | 3.795 | 3.387 | 0.408 | 2.528 | 0.012 |
| IMQ26: Were you in suspense about whether or not you would win or lose the game? | 3.295 | 2.669 | 0.626 | 3.715 | 0.000 |
| IMQ27: At any point did you find yourself become so involved that you wanted to speak to the game directly? | 1.992 | 1.750 | 0.242 | 1.558 | 0.121 |
| IMQ28: To what extent did you enjoy the graphics and the imagery? | 3.443 | 3.081 | 0.362 | 2.253 | 0.025 |
| IMQ29: How much would you say you enjoyed playing the game? | 3.221 | 3.008 | 0.213 | 1.316 | 0.190 |
| IMQ30: When interrupted, were you disappointed that the game was over? | 2.623 | 2.387 | 0.236 | 1.306 | 0.193 |
| IMQ31: Would you like to play the game again? | 2.951 | 2.710 | 0.241 | 1.360 | 0.175 |

^*^ *p* < 0.05, ^**^ *p* < 0.01, ^***^ *p* < 0.001
